# Supplementary material for: Oncolytic effects of the recombinant Newcastle disease virus, rAF-IL12, against colon cancer cells in vitro and in tumor-challenged NCr-Foxn1nu nude mice
Source: PeerJ. 2020 Dec 8;8:e9761. doi: 10.7717/peerj.9761 (PMC7731658; doi:10.7717/peerj.9761)
Supplement: Supplemental Information 1 [file peerj-08-9761-s001.docx]

**Table S1. Percentage rate of survival of HT29 tumor-burdened nude mice from the negative control, AF2240-i-treated, and rAF-IL12-treated groups starting from day-0 until day-28 of treatment.**

| **Group** | **Total number of mice at day-0** | **Total number of mice at day-28** | **Percentage rate of survival (%)** |
| --- | --- | --- | --- |
| Negative Control | 6/6 | 6/6 | 100 |
| AF2240-i | 6/6 | 6/6 | 100 |
| rAF-IL12 | 6/6 | 6/6 | 100 |
